# Supplementary material for: Virtual Screening and Molecular Dynamics Simulation Study of Influenza Polymerase PB2 Inhibitors
Source: Molecules. 2021 Nov 17;26(22):6944. doi: 10.3390/molecules26226944 (PMC8623395; doi:10.3390/molecules26226944)
Supplement: Supplementary file 1 [file molecules-26-06944-s001.zip › molecules-1416548-supplementary.pdf]

Virtual screening and molecular dynamics simulation study of influenza polymerase PB2 inhibitors

Supporting information

Verification of docking method

Figure S1 Comparison between the highest ranked pose of VX-787 and the original pose in the crystal structur. The original pose was presented as green stick, the predicted highest ranking pose was presented as blue stick.

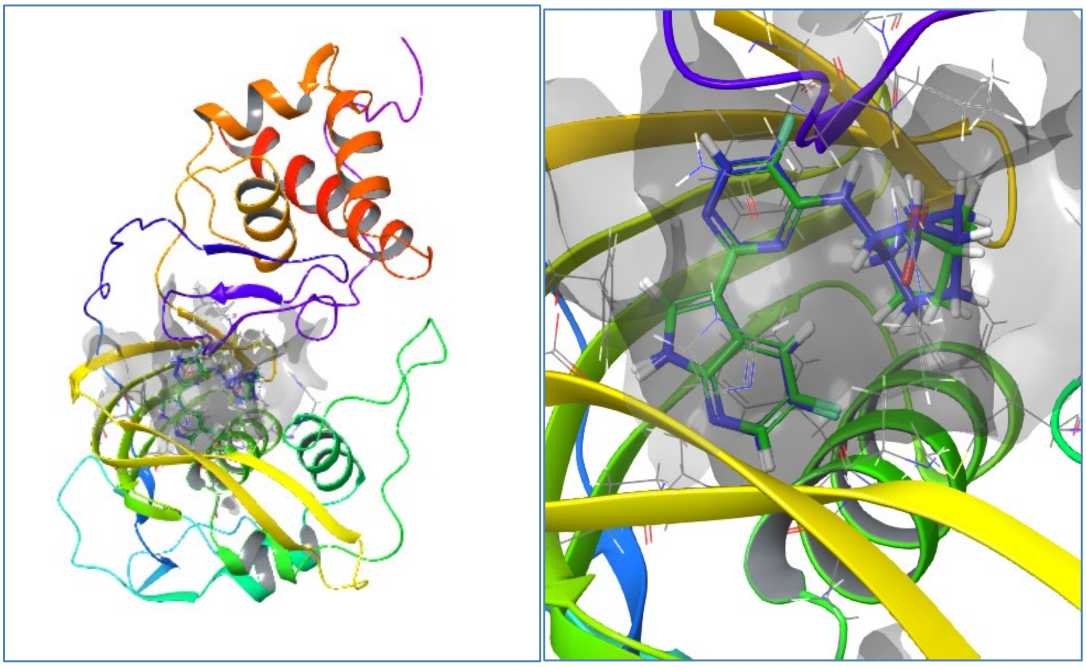

Figure S2. The Structure, ID, XP glidescore, clusters and predicted absorption level and solubility level of compounds retained by VSW.

| | | |
|---|---|---|
| 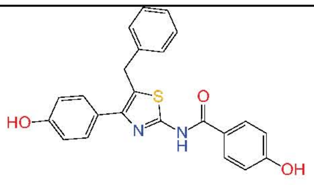<br>title: Str1595<br>XP GScore: 8.017<br>ADMET Absorption Level: 1<br>ADMET Solubility Level: 2<br>Cluster: 1 | 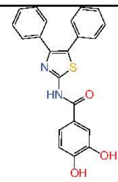<br>title: Str1546<br>XP GScore: 8.509<br>ADMET Absorption Level: 1<br>ADMET Solubility Level: 2<br>Cluster: 1 | 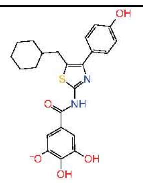<br>title: Str1604<br>XP GScore: 7.869<br>ADMET Absorption Level: 2<br>ADMET Solubility Level: 2<br>Cluster: 1 |
| 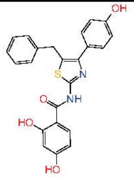<br>title: Str1586<br>XP GScore: 8.759<br>ADMET Absorption Level: 1<br>ADMET Solubility Level: 2<br>Cluster: 1 | 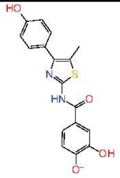<br>title: Str1606<br>XP GScore: 8.902<br>ADMET Absorption Level: 0<br>ADMET Solubility Level: 3<br>Cluster: 1 | 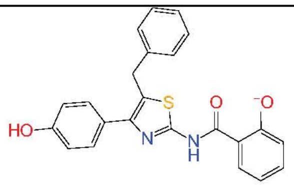<br>title: Str1587<br>XP GScore: 8.914<br>ADMET Absorption Level: 0<br>ADMET Solubility Level: 2<br>Cluster: 1 |
| 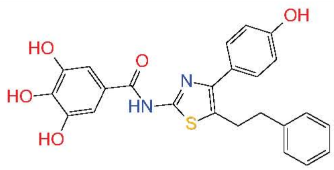<br>title: Str1614<br>XP GScore: 9.321<br>ADMET Absorption Level: 2<br>ADMET Solubility Level: 2<br>Cluster: 1 | 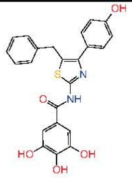<br>title: Str1589<br>XP GScore: 9.641<br>ADMET Absorption Level: 2<br>ADMET Solubility Level: 2<br>Cluster: 1 | 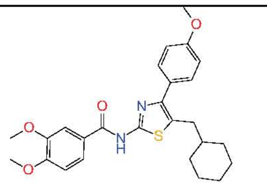<br>title: Str1597<br>XP GScore: 7.052<br>ADMET Absorption Level: 2<br>ADMET Solubility Level: 1<br>Cluster: 1 |
| 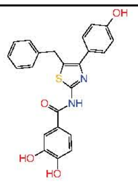<br>title: Str1569<br>XP GScore: 10.8<br>ADMET Absorption Level: 1<br>ADMET Solubility Level: 2<br>Cluster: 1 | 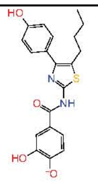<br>title: Str1616<br>XP GScore: 9.854<br>ADMET Absorption Level: 0<br>ADMET Solubility Level: 2<br>Cluster: 1 | 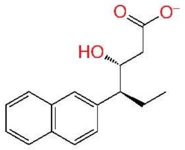<br>title: Str8061<br>XP GScore: 8.276<br>ADMET Absorption Level: 0<br>ADMET Solubility Level: 3<br>Cluster: 2 |
| 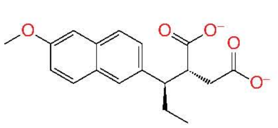<br>title: Str7418<br>XP GScore: 8.078<br>ADMET Absorption Level: 0<br>ADMET Solubility Level: 4<br>Cluster: 2 | 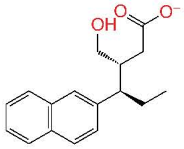<br>title: Str5903<br>XP GScore: 8.762<br>ADMET Absorption Level: 0<br>ADMET Solubility Level: 3<br>Cluster: 2 | 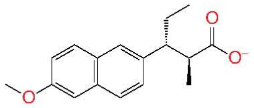<br>title: Str1056<br>XP GScore: 7.84<br>ADMET Absorption Level: 0<br>ADMET Solubility Level: 3<br>Cluster: 2 |

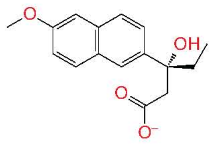

title: Str5757
XP GScore: 7.226
ADMET Absorption Level: 0
ADMET Solubility Level: 3
Cluster: 2

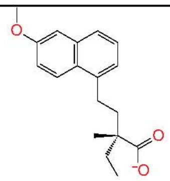

title: Str1054
XP GScore: 7.539
ADMET Absorption Level: 0
ADMET Solubility Level: 2
Cluster: 2

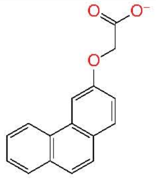

title: Str7337
XP GScore: 7.503
ADMET Absorption Level: 0
ADMET Solubility Level: 3
Cluster: 2

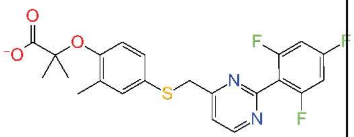

title: Str2169
XP GScore: 7.279
ADMET Absorption Level: 0
ADMET Solubility Level: 2
Cluster: 3

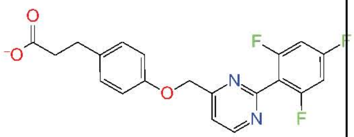

title: Str2165
XP GScore: 7.636
ADMET Absorption Level: 0
ADMET Solubility Level: 3
Cluster: 3

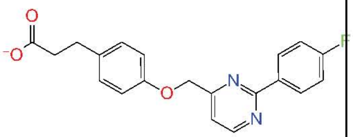

title: Str2157
XP GScore: 7.195
ADMET Absorption Level: 0
ADMET Solubility Level: 3
Cluster: 3

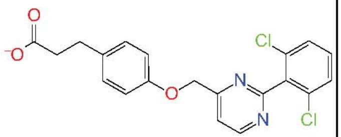

title: Str2163
XP GScore: 7.38
ADMET Absorption Level: 0
ADMET Solubility Level: 2
Cluster: 3

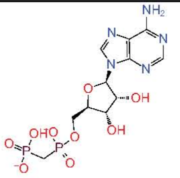

title: Str921
XP GScore: 8.241
ADMET Absorption Level: 3
ADMET Solubility Level: 4
Cluster: 4

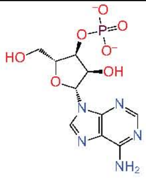

title: Str922
XP GScore: 8.39
ADMET Absorption Level: 3
ADMET Solubility Level: 4
Cluster: 4

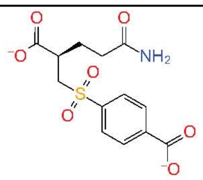

title: Str1105
XP GScore: 7.745
ADMET Absorption Level: 3
ADMET Solubility Level: 5
Cluster: 5

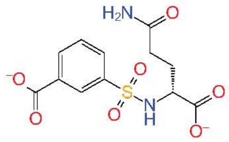

title: Str1107
XP GScore: 8.252
ADMET Absorption Level: 3
ADMET Solubility Level: 5
Cluster: 5

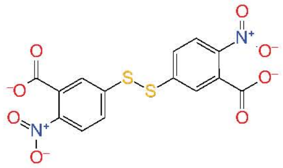

title: Str6530
XP GScore: 7.296
ADMET Absorption Level: 3
ADMET Solubility Level: 3
Cluster: 6

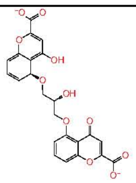

title: Str4660
XP GScore: 7.437
ADMET Absorption Level: 3
ADMET Solubility Level: 5
Cluster: 7

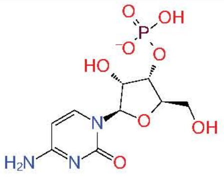

title: Str924
XP GScore: 8.951
ADMET Absorption Level: 3
ADMET Solubility Level: 4
Cluster: 8

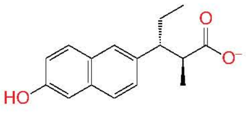

title: Str5748
XP GScore: 8.365
ADMET Absorption Level: 0
ADMET Solubility Level: 3
Cluster: 9

| | | |
|---|---|---|
| 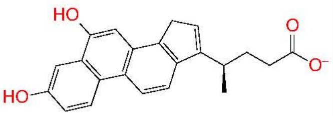<br>title: Str7750<br>XP GScore: 8.436<br>ADMET Absorption Level: 0<br>ADMET Solubility Level: 2<br>Cluster: 9 | 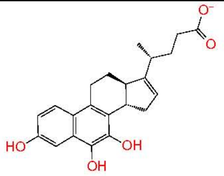<br>title: Str7749<br>XP GScore: 9.726<br>ADMET Absorption Level: 0<br>ADMET Solubility Level: 3<br>Cluster: 9 | 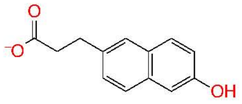<br>title: Str1062<br>XP GScore: 7.891<br>ADMET Absorption Level: 0<br>ADMET Solubility Level: 3<br>Cluster: 9 |
| 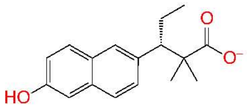<br>title: Str4425<br>XP GScore: 8.606<br>ADMET Absorption Level: 0<br>ADMET Solubility Level: 3<br>Cluster: 9 | 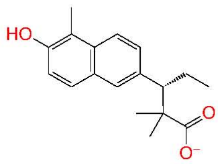<br>title: Str5751<br>XP GScore: 8.904<br>ADMET Absorption Level: 0<br>ADMET Solubility Level: 2<br>Cluster: 9 | 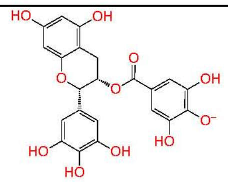<br>title: Str317<br>XP GScore: 11.015<br>ADMET Absorption Level: 3<br>ADMET Solubility Level: 2<br>Cluster: 10 |
| 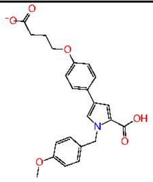<br>title: Str806<br>XP GScore: 7.893<br>ADMET Absorption Level: 0<br>ADMET Solubility Level: 3<br>Cluster: 11 | 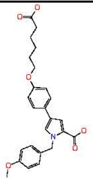<br>title: Str807<br>XP GScore: 7.992<br>ADMET Absorption Level: 0<br>ADMET Solubility Level: 3<br>Cluster: 11 | 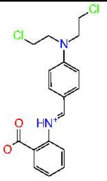<br>title: Str3839<br>XP GScore: 7.327<br>ADMET Absorption Level: 0<br>ADMET Solubility Level: 3<br>Cluster: 12 |
| 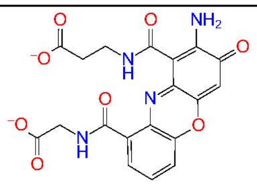<br>title: Str6017<br>XP GScore: 8.932<br>ADMET Absorption Level: 3<br>ADMET Solubility Level: 5<br>Cluster: 13 | 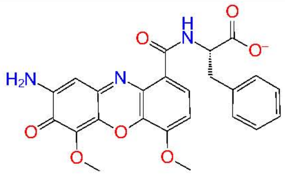<br>title: Str7374<br>XP GScore: 8.511<br>ADMET Absorption Level: 2<br>ADMET Solubility Level: 3<br>Cluster: 13 | 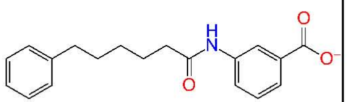<br>title: Str6317<br>XP GScore: 8.131<br>ADMET Absorption Level: 0<br>ADMET Solubility Level: 3<br>Cluster: 14 |
| 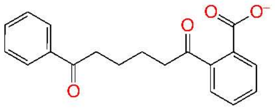<br>title: Str6318<br>XP GScore: 7.389<br>ADMET Absorption Level: 0<br>ADMET Solubility Level: 3<br>Cluster: 14 | 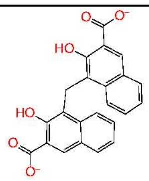<br>title: Str6842<br>XP GScore: 9.467<br>ADMET Absorption Level: 0<br>ADMET Solubility Level: 3<br>Cluster: 15 | 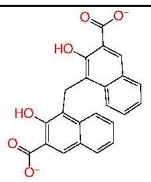<br>title: Str3107<br>XP GScore: 9.993<br>ADMET Absorption Level: 0<br>ADMET Solubility Level: 3<br>Cluster: 15 |

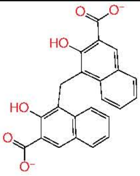

title: Str3952
XP GScore: 8.874
ADMET Absorption Level: 0
ADMET Solubility Level: 3
Cluster: 15

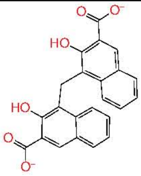

title: Str5621
XP GScore: 8.882
ADMET Absorption Level: 0
ADMET Solubility Level: 3
Cluster: 15

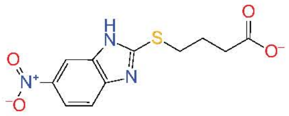

title: Str7642
XP GScore: 7.046
ADMET Absorption Level: 0
ADMET Solubility Level: 3
Cluster: 16

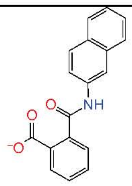

title: Str6038
XP GScore: 7.494
ADMET Absorption Level: 0
ADMET Solubility Level: 3
Cluster: 17

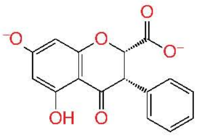

title: Str7486
XP GScore: 7.888
ADMET Absorption Level: 0
ADMET Solubility Level: 4
Cluster: 18

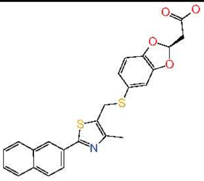

title: Str1748
XP GScore: 7.143
ADMET Absorption Level: 0
ADMET Solubility Level: 2
Cluster: 19

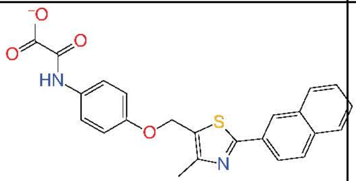

title: Str1760
XP GScore: 7.683
ADMET Absorption Level: 0
ADMET Solubility Level: 2
Cluster: 19

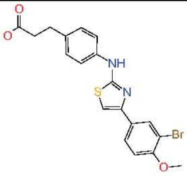

title: Str1902
XP GScore: 8.191
ADMET Absorption Level: 0
ADMET Solubility Level: 2
Cluster: 20

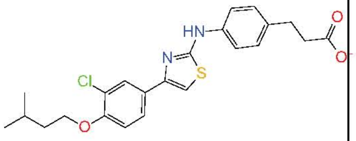

title: Str1919
XP GScore: 8.14
ADMET Absorption Level: 0
ADMET Solubility Level: 2
Cluster: 20

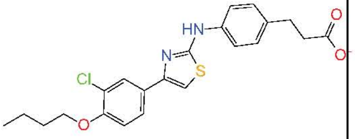

title: Str1916
XP GScore: 8.115
ADMET Absorption Level: 0
ADMET Solubility Level: 2
Cluster: 20

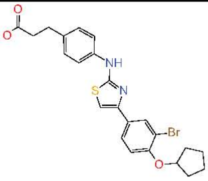

title: Str1905
XP GScore: 8.413
ADMET Absorption Level: 0
ADMET Solubility Level: 1
Cluster: 20

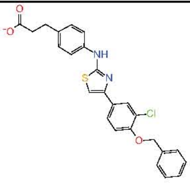

title: Str1921
XP GScore: 8.439
ADMET Absorption Level: 0
ADMET Solubility Level: 2
Cluster: 20

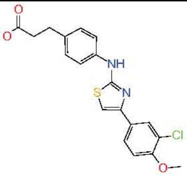

title: Str1912
XP GScore: 7.818
ADMET Absorption Level: 0
ADMET Solubility Level: 2
Cluster: 20

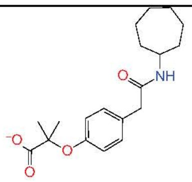

title: Str260
XP GScore: 7.453
ADMET Absorption Level: 0
ADMET Solubility Level: 3
Cluster: 21

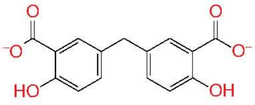

title: Str8015
XP GScore: 9.021
ADMET Absorption Level: 0
ADMET Solubility Level: 4
Cluster: 22

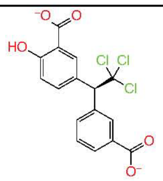

title: Str5776
XP GScore: 8.569
ADMET Absorption Level: 0
ADMET Solubility Level: 3
Cluster: 22

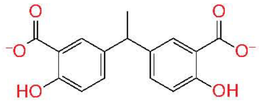

title: Str5760
XP GScore: 9.728
ADMET Absorption Level: 0
ADMET Solubility Level: 4
Cluster: 22

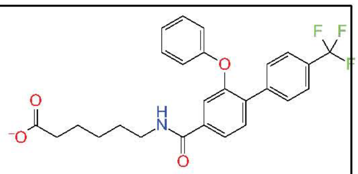

title: Str613
XP GScore: 8.004
ADMET Absorption Level: 0
ADMET Solubility Level: 2
Cluster: 23

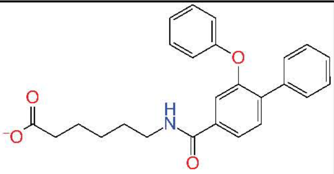

title: Str611
XP GScore: 9.486
ADMET Absorption Level: 0
ADMET Solubility Level: 2
Cluster: 23

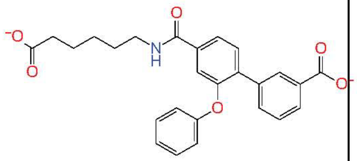

title: Str618
XP GScore: 9.752
ADMET Absorption Level: 0
ADMET Solubility Level: 3
Cluster: 23

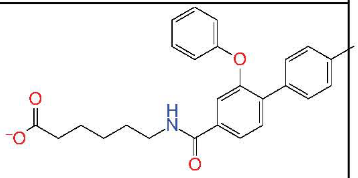

title: Str612
XP GScore: 8.966
ADMET Absorption Level: 0
ADMET Solubility Level: 2
Cluster: 23

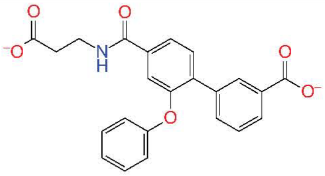

title: Str627
XP GScore: 8.894
ADMET Absorption Level: 0
ADMET Solubility Level: 3
Cluster: 23

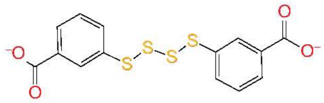

title: Str6666
XP GScore: 7.19
ADMET Absorption Level: 0
ADMET Solubility Level: 3
Cluster: 24

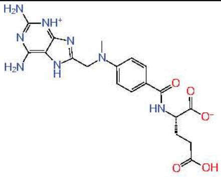

title: Str6860
XP GScore: 7.583
ADMET Absorption Level: 3
ADMET Solubility Level: 3
Cluster: 25

Figure S3. The structure, ID, XP glidescore, clusters, and predicted absorption levels and solubility levels of compounds selected for biological evaluation.

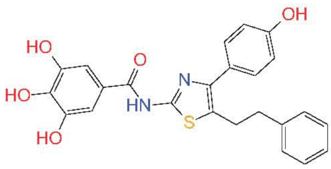

title: Str1614
XP GScore: 9.321
ADMET Absorption Level: 2
ADMET Solubility Level: 2
Cluster: 1

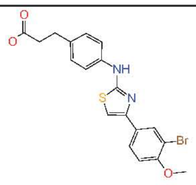

title: Str1902
XP GScore: 8.191
ADMET Absorption Level: 0
ADMET Solubility Level: 2
Cluster: 20

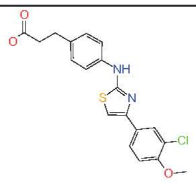

title: Str1912
XP GScore: 7.818
ADMET Absorption Level: 0
ADMET Solubility Level: 2
Cluster: 20

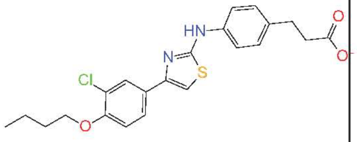

title: Str1916
XP GScore: 8.115
ADMET Absorption Level: 0
ADMET Solubility Level: 2
Cluster: 20

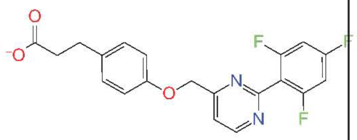

title: Str2165
XP GScore: 7.636
ADMET Absorption Level: 0
ADMET Solubility Level: 3
Cluster: 3

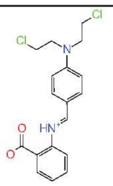

title: Str3839
XP GScore: 7.327
ADMET Absorption Level: 0
ADMET Solubility Level: 3
Cluster: 12

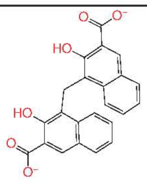

title: Str3952
XP GScore: 8.874
ADMET Absorption Level: 0
ADMET Solubility Level: 3
Cluster: 15

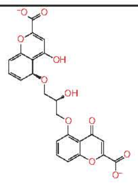

title: Str4660
XP GScore: 7.437
ADMET Absorption Level: 3
ADMET Solubility Level: 5
Cluster: 7

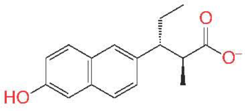

title: Str5748
XP GScore: 8.365
ADMET Absorption Level: 0
ADMET Solubility Level: 3
Cluster: 9

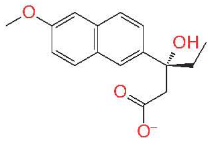

title: Str5757
XP GScore: 7.226
ADMET Absorption Level: 0
ADMET Solubility Level: 3
Cluster: 2

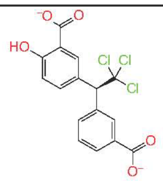

title: Str5776
XP GScore: 8.569
ADMET Absorption Level: 0
ADMET Solubility Level: 3
Cluster: 22

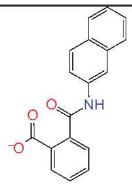

title: Str6038
XP GScore: 7.494
ADMET Absorption Level: 0
ADMET Solubility Level: 3
Cluster: 17

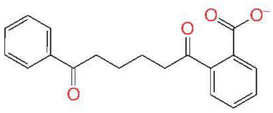

title: Str6318
XP GScore: 7.389
ADMET Absorption Level: 0
ADMET Solubility Level: 3
Cluster: 14

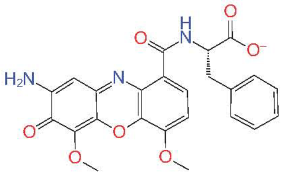

title: Str7374
XP GScore: 8.511
ADMET Absorption Level: 2
ADMET Solubility Level: 3
Cluster: 13

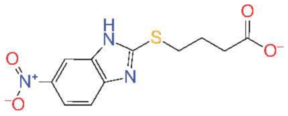

title: Str7642
XP GScore: 7.046
ADMET Absorption Level: 0
ADMET Solubility Level: 3
Cluster: 16

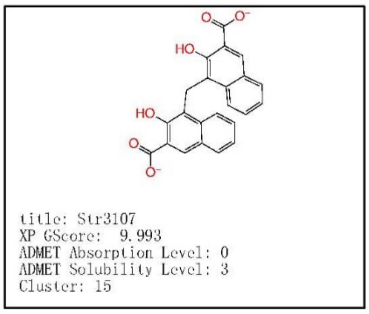


Figure S4. The RMSD of PB2 protein Cα atoms and Lig fit Prot of Ligand in MD simulations.

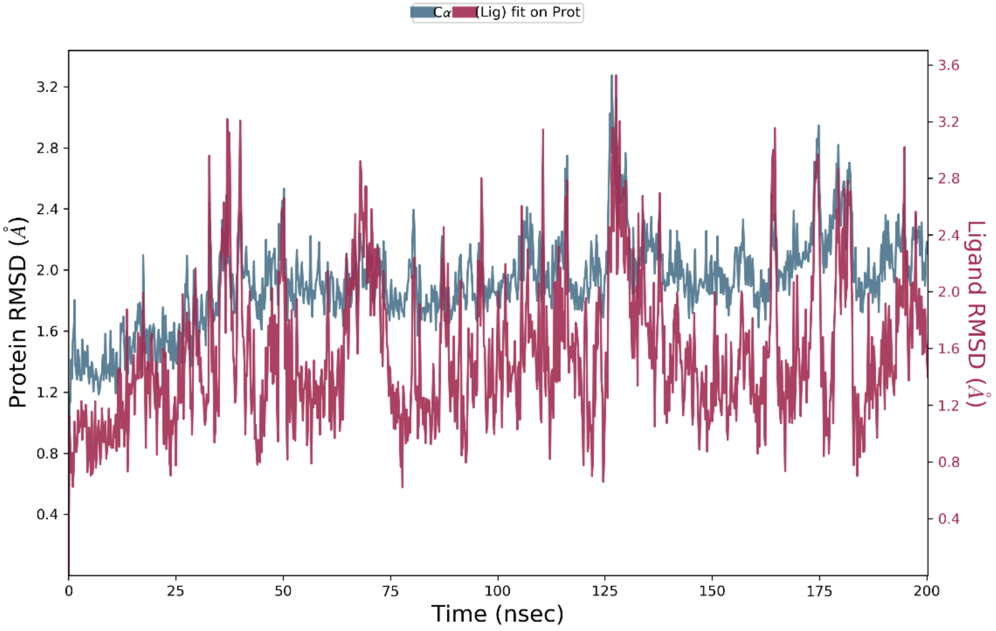


VX-787

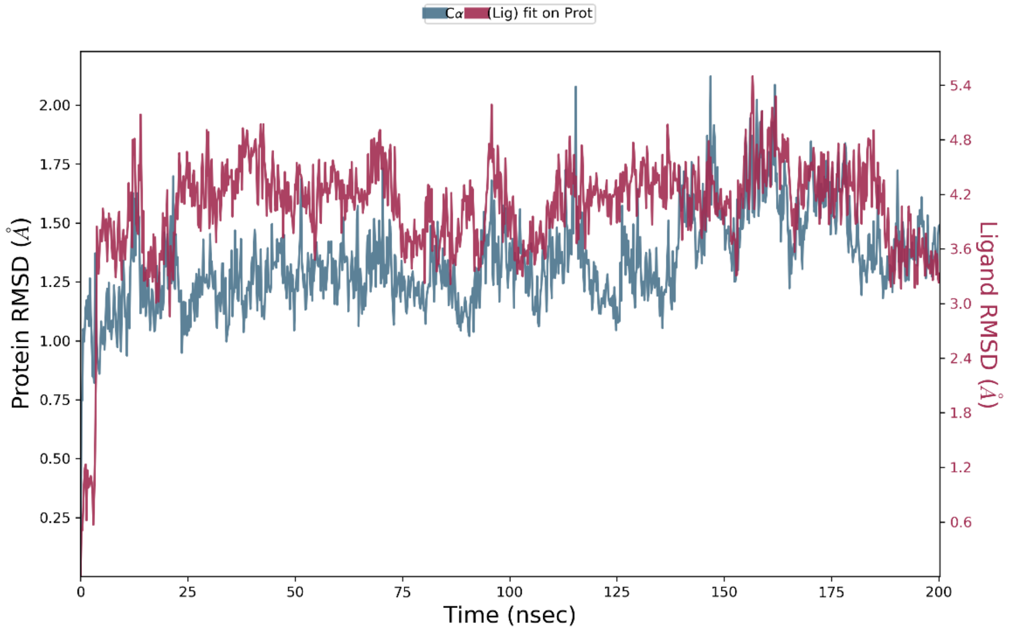


STR1614

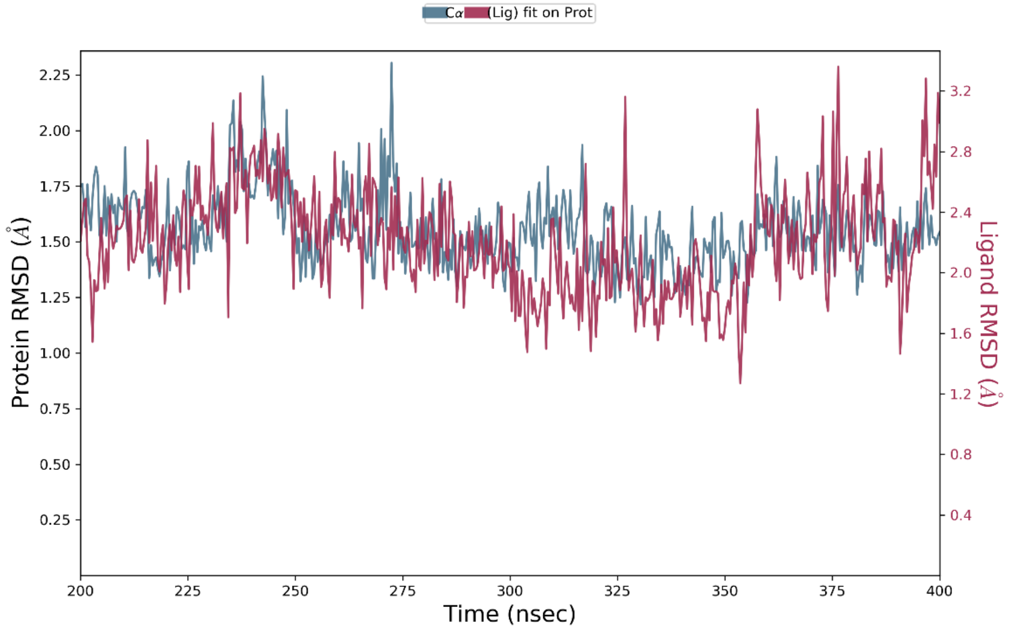


STR1916

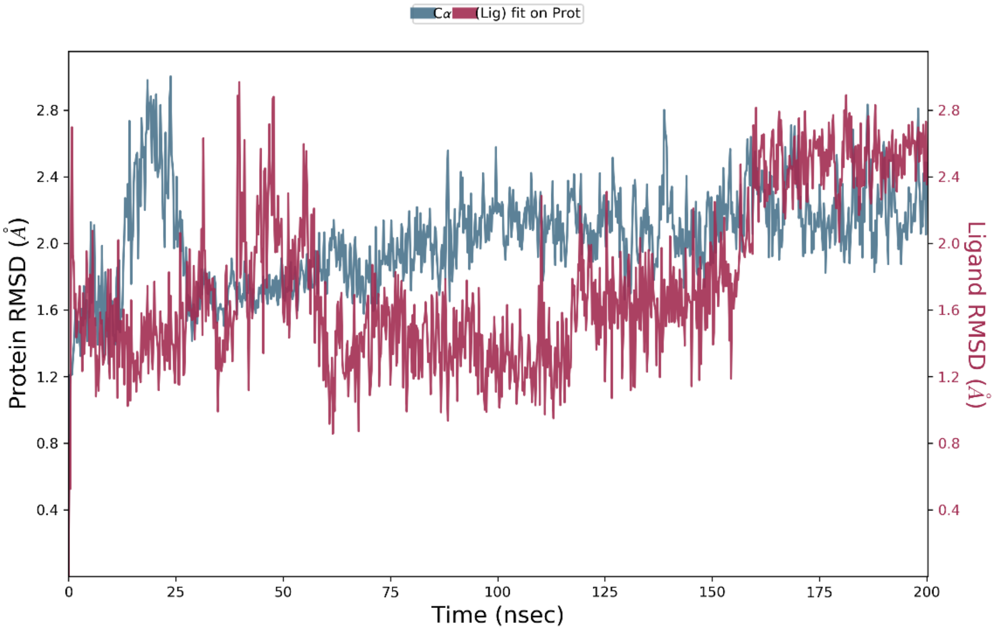


STR3107

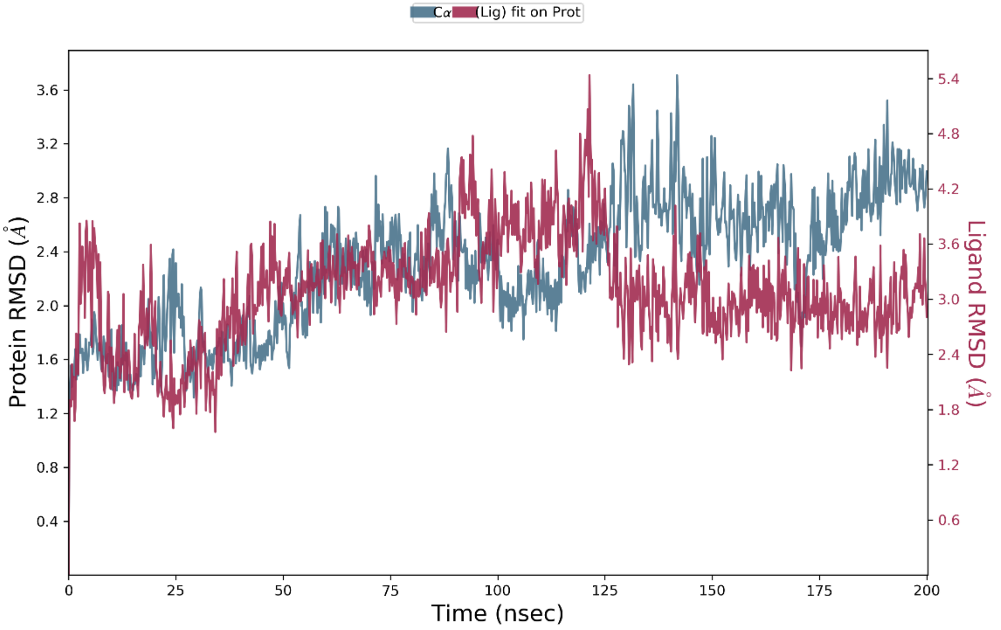


STR5776

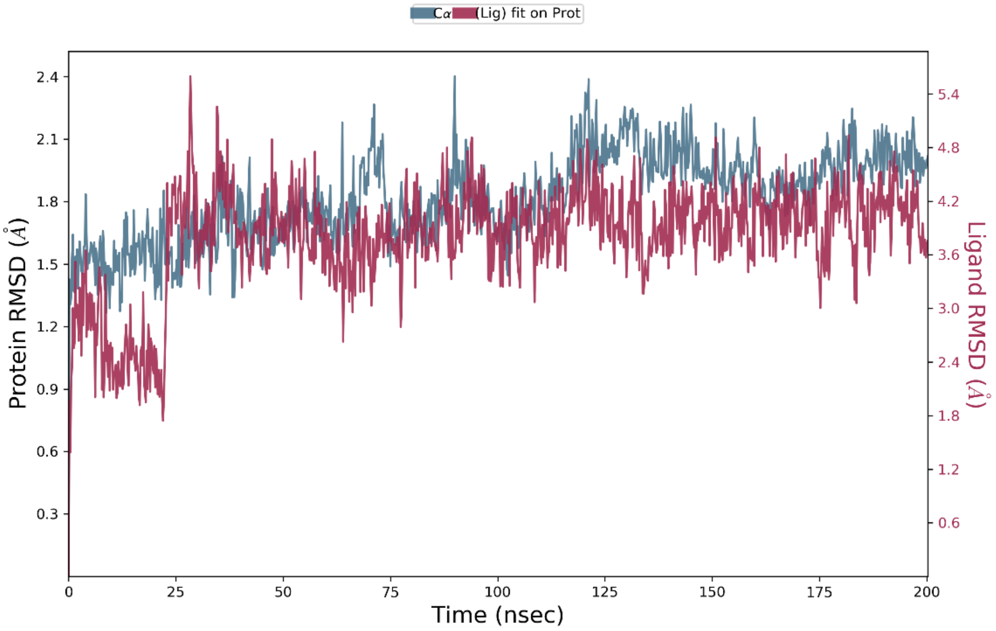


Str6318

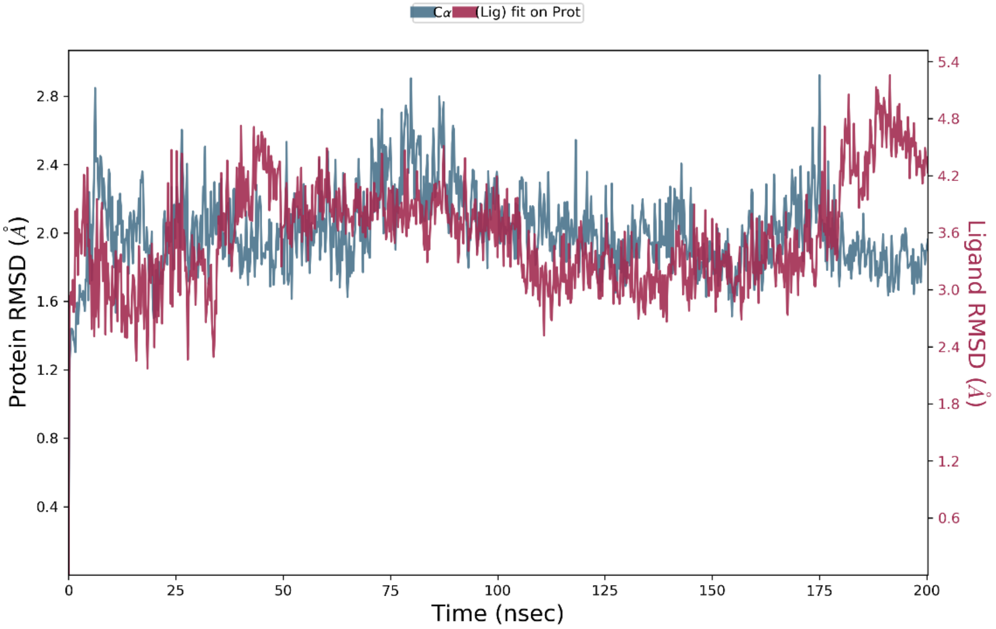


STR7374
